# Causes of Fever and Ague

**Published:** 1876-10

**Authors:** 


*TWENTY-THIRD YEAR OF*

### HALL'S JOURNAL OF HEALTH.

***E. H. Gibbs, M.D., Editor.***

VOL. XXIII.] OCTOBER, 1876. [NO. 10.

### *CAUSES OF FEVER AND AGUE.*

There has long existed a diversity of opinion in regard to the principal causes of intermittent fever. Many years ago it was generally supposed to be produced by the malaria of marshy districts, and a residence in their vicinity was considered hazardous. We will not say that the malaria from marshes has nothing to do with intermittent and other fevers ; but we do not believe it is the chief cause of their development.

We are acquainted with many families that have lived for years on the very borders of marsh lands, and have enjoyed perfect health all the time, while the residents of the high lands near them, have suffered greatly from fevers.

We are somewhat skeptical as to the power of miasm arising from natural marshes, to produce disease. Where the water of mill ponds is drawn out during the day, and a mass of vegetable matter is exposed to the heat of the sun, or where large masses of earth are thrown up from considerable depth below the surface, sickness is almost certain to prevail in the neighborhood, and often extends for miles around. Bating these causes of fever, which causes are entirely artificial, we do not believe that locality has much to do in producing them. Imprudence in dress, in diet, and in the habits of life generally, will sooner or later bring on disease ; a little sooner, perhaps, in localities where the climate is not perfectly salubrious, since another element is added to those that already exist.

He who expects to enjoy immunity from illness by simply selecting a healthy place of residence without the exercise of prudence in living, will be disappointed. There is no doubt that the principal cause of nearly every form of fever and of many other diseases, is impure water.

The second in order is the proximity to cess-pools and decaying animal and vegetable matter. Third—exposure to chilly air of the evening and morning. Fourth—over exertion and insufficient sleep and rest. Fifth—want of attention to the condition of the bowels.

The first essential to health is an ample supply of pure fresh water. No family can afford to do without this. Any expenditure necessary to procure it, will prove to be the very best economy. Springs and wells should be protected against the entrance of surface water, which is contaminated with the filth of the soil through which it percolates.

Such water can not, for any length of time, be taken into the system without producing disease, regardless of climate or location. "Driven-wells" are the best and safest. But there are localities where it is not possible to have good wells. In that case cisterns should be constructed with filters to keep out all impurities.

In places where neither wells nor cisterns are practicable, all drinking water should be boiled or distilled. It is probably safe to say, that more than half of the water used for drinking is unwholesome and dangerous to health.

There are but few persons who will dissent from these views. But when the question of diet is discussed all harmony of opinion ceases. Almost every man has his peculiar theory. It is wonderful that there should be such diversity of ideas over such a simple problem. Some strive to live on the "smallest modicum of alimentary matter," and look upon all men as gluttons who do not do likewise.

It does not seem to occur to the advocates of low diet, that the system requires a certain amount of strong, wholesome food to enable it to live, and that nature dictates unerringly what that quantity shall be, regardless of all theories to the contrary.

The quality of food is more important than the quantity. If a man lives upon food that contains but little nutriment, he only lives; he has but little strength or heart for the active duties of life. Going out into the open air just at evening or early in the morning, without protecting the body with sufficient clothing, is always hazardous. Becoming frequently overfatigued is apt to result in debility, and debility is disease. The last, but not the least important cause of disease that we have referred to above, is want of attention to the condition of the bowels.

No parent does his whole duty who neglects to look after the health of his children, and there is nothing more important for them than that they should be regular in this respect. It may save them years of misery and early death, from some disease certain to be induced by habitual constipation. Nothing will so effectually protect a person against fevers and all other diseases as regularity of the bowels. We wonder that this trouble is so prevalent, since it may be easily and permanently remedied in almost every instance. Nearly all diseases are contracted through carelessness or ignorance, and they are allowed to become chronic and to wear away life by neglect of timely and proper treatment.

